# Comparison of clinical outcome between stereotactic body radiotherapy and radiofrequency ablation for unresectable hepatocellular carcinoma

**DOI:** 10.1097/MD.0000000000028545

**Published:** 2022-01-28

**Authors:** Ren Ji, Kelvin K. Ng, Wenqi Chen, Weihong Yang, Hongtao Zhu, Tan-To Cheung, Chi-Leung Chiang, Tiffany C.L. Wong, Feng-Ming Kong, G. Wu, Chung-Mau Lo

**Affiliations:** a Division of Hepato-Biliary and Pancreatic Surgery, Department of Surgery, The University of Hong Kong-Shenzhen Hospital, China; b Department of Clinical Oncology, The University of Hong Kong-Shenzhen Hospital, China; c Division of Interventional Radiology, Department of Medical Imaging, The University of Hong Kong-Shenzhen Hospital, China; d Department of Clinical Oncology, Li Ka Shing Faculty of Medicine, The University of Hong Kong, Hong Kong, China.

**Keywords:** body, carcinoma, hepatocellular, radiotherapy, stereotactic

## Abstract

Stereotactic body radiotherapy (SBRT) is a novel noninvasive treatment for unresectable hepatocellular carcinoma (HCC). Whether its efficacy is comparable to radiofrequency ablation (RFA), a recommended therapy for unresectable HCC, is unknown. The present study aims to compare the clinical outcome between SBRT and RFA for patients with unresectable HCC.

The clinical data of 60 patients with unresectable HCC from January 2018 to January 2021 were retrospectively reviewed. There were 22 cases treated by SBRT and 38 cases by RFA. The short-term and long-term clinical outcomes were compared.

There was no significant difference in the baseline demographic characteristics between two groups. The complete remission rate at 3 months was comparable between SBRT group (81.8%) and RFA group (89.4%). Local tumor control rate was also similar between two groups (90.9% vs. 94.7%). There was no severe complication (grade IIIa or above) in both groups. The 1-year and 2-year overall survival rates were 88.2% and 85.7% in SBRT group and 100% and 75% in RFA group, respectively. There was no statistical significant difference between groups (*P* = .576).

SBRT can achieve similar short and long-term clinical outcome as RFA for unresectable HCC. Future prospective clinical study is needed to justify its role in patients with HCC.

## 1 Introduction

Hepatocellular carcinoma (HCC) is currently the sixth most common malignant tumor and the third leading cause of cancer death in the world in 2020.^[1]^ Hepatic resection offers the best treatment option for HCC with favorable 5-year overall survival rate up to 60%.^[2,3]^ However, the resectability rate patients with HCC remains low (about 20%) because of multicentric nature of tumor, unsatisfactory liver function, and propensity vascular invasion to portal venous system by tumor. Local ablation therapy using radiofrequency ablation (RFA) offers another viable treatment option for unresectable HCC.^[4,5]^ Even after curative hepatic resection, intrahepatic tumor recurrence is common (up to 50%–70%) because of intrahepatic metastasis through portal venous system and de novo tumor from the underlying hepatitis viral infection.^[6]^ It will then be preferable that local ablation therapy can be applied to these recurrent tumor if feasible.

The application of RFA for liver tumor is limited by difficult locations of tumors (dome of liver, subcapsular region in close proximity to internal organs and perivascular location), which will preclude the route of percutaneous approach for insertion of RFA needle. In other words, either laparoscopic or open approach is needed in those situations, which carry significant surgical trauma. Recently, stereotactic body radiotherapy (SBRT) has evolved as a new local ablation therapy for HCC, which is totally noninvasive.^[7]^ It is external beam radiation therapy which is delivered in hypofractionated manner with high energy dose in each fraction by the use of advanced radiation planning and delivery. Early phase clinical trials of SBRT has proven its efficacy for HCC, with local tumor control rate up to 99% at follow-up period up to 3 years.^[8,9]^ Over the years, there has been limited retrospective studies in the literature comparing SBRT and RFA regarding the clinical efficacy for unresectable HCC.^[9–11]^ The reported short-term and mid-term outcome of SBRT were comparable to RFA. Moreover, SBRT has been shown to be effective for large HCC (>3 cm) at difficult anatomical location.^[11]^ Lately, meta-analysis studies even showed that SBRT had better local tumor control than RFA, although long-term overall survival results of SBRT was conflicting.^[12,13]^ To solidify the treatment role of SBRT for HCC, a retrospective comparative study was conducted in authors’ center. The present study aims to compare the clinical outcome between SBRT and RFA for patients with unresectable HCC, in terms of perioperative outcome, local tumor control, and long-term survival.

## 2 Patients and methods

This study has been approved by institutional review broad of The University of Hong Kong-Shenzhen Hospital.

From January 2018 to January 2021, there were 60 patients with unresectable HCC who were treated by SBRT (n = 22) or RFA (n = 38) in the Department of Surgery, The University of Hong Kong-Shenzhen Hospital. The inclusion criteria for SBRT or RFA was as follows:

(a) The diagnosis of HCC followed the criteria used by the European Association for the Study of the Liver.^[4]^ HCC was diagnosed when the radiologic imaging techniques (spiral contrasted computer tomography [CT] scan or contrasted magnetic resonance imaging [MRI]) showed typical features of HCC (contrast enhancement in the arterial phase and rapid wash-out of contrast in the venous or delayed phase) and/or the serum alpha fetoprotein (AFP) level was elevated (>400 ηg/mL).
(b) Unresectable HCC is due to poor liver function (Child-Pugh grade B or indocyanine green retention test >20% at 15 min in case of major hepatectomy).
(c) Tumor size ≤ 5 cm and/or no. of tumors ≤3.
(d) Absence of portal vein invasion.
(e) Absence of extrahepatic metastasis.
(f) Liver function status of Child-Pugh grade A or B.

The exclusion criteria included

(a) patients with tumor invasion to major intrahepatic vasculature (portal vein and/or hepatic vein branches),
(b) patients with extrahepatic tumor metastasis, and
(c) patients with liver function status of Child-Pugh grade C.

For all patients in both groups, one dose of transarterial chemoembolization was given before SBRT or RFA. Antiviral medication (Entecavir 0.5 mg daily po) was prescribed to all hepatitis B carriers in both groups.

### 2.1 Stereotactic body radiotherapy

SBRT system (*Varian Company, America*) was adopted for all patients in this group. A 4D – CT technology was also used to determine optimal treatment areas of target lesion. To enhance accuracy of tumor targeting, the breathing movement of patients was kept minimal (amplitude of liver movement was controlled within 5 mm). All patients received breathing exercise training to practice shallow breathing before the procedure. Two experienced intervention radiologists examined the gross tumor volume and the planning target volume of tumors. In selected cases, the irradiation field was adjusted by 50% to 60% of original prescription isodose curve surrounding planning target volume to avoid collateral organ damage. In general, the irradiation planning would be 5.5 to 10 Gy per day for 5 doses in 1 week. The total irradiation dose would be 27.5 to 50 Gy for each patient.

### 2.2 Radiofrequency ablation

All patients received RFA through percutaneous approach under ultrasound or CT guidance. The procedure adopt the Cool-tip radiofrequency system (Radionics, Burlington, MA). A single RF needle with an exposed length of 3 cm was used for tumor ≤3 cm in diameter, whereas a clustered needle (three parallel single needle close to each other) with an exposed length of 2.5 cm was used to treat large tumors >3 cm. Patients were under monitored anesthetic control. Each RFA ablation cycle lasted for 8 to 12 min, and multiple overlapping ablation zones were required in large tumors. Upon completion of RFA procedure, needle track ablation was carried out to avoid tumor seeding along the needle track.

### 2.3 Data collection and outcome measures

Clinical data of all patients were collected in a database. The clinical details, short-term and long-term outcome measures were retrospectively evaluated in all 60 patients. The short-term outcome measures include post-procedure complication, treatment-related mortality, and complete remission (CR) rate at 3 months as measured by CT scan or MRI at 3 months after the procedure. A complication was defined as any adverse event after the procedure according to Clavien-Dindo classification.^[14]^ Treatment-related mortality was defined as any death within 30 days after the procedure. All patients had monitoring of serum AFP level, chest radiograph, and CT scan/MRI every 3 to 4 months after the procedure. Local tumor response was evaluated according to the modified Response Evaluation Criteria in Solid Tumors formulated by the American Association for the Study of Liver Diseases.^[15]^ CR referred to the disappearance of the arterial contrast enhancement of all target lesions and this referred to complete local tumor control. Partial response referred to the reduction of the total diameter of the target lesion by ≥30% in the arterial contrast enhancement. Stable disease referred to the diameter of the lesion reduced by <30% or increased by <20%. Progressive disease referred to the increase in the diameter of the target lesion by ≥20%, or the appearance of a new lesion. Complete ablation rate of tumor referred to CR on imaging studies at 3 months after treatment. Local tumor control rate referred to CR, partial response, and stable disease on follow-up imaging studies.

### 2.4 Statistical analysis

Continuous data were expressed as median with ranges and were compared using Mann–Whitney *U* test. Categorical data were compared using the Chi square test with Yates’ correction or the Fisher's exact test where appropriate. The overall and disease-free survival rates were calculated by the Kaplan–Meier method and compared using the log-rank test. Hospital deaths were included in the overall survival analysis but were excluded from the disease-free survival analysis. All statistical analyses were performed using a statistical software (SPSS 25.0 for Windows, SPSS Inc., Chicago, IL). A *P-*value of less than .05 was considered statistically significant.

## 3 Results

### 3.1 Patient characteristics

Among 22 patients in SBRT group and 38 patients in RFA groups, there was no statistical significant difference in the baseline patient demographic characteristics, including age, gender, proportion of hepatic B carriers, presence of cirrhosis, comorbidity, and liver function in terms of liver biochemistry, Child-Pugh Grading, and Indocyanine Green – 15.

In terms of tumor characteristics, SBRT group has significantly larger tumor than RFA group, but the total tumor number was similar between two groups. Majority of patients in SBRT group had HCC at difficult anatomical locations (liver dome, n = 5; perivascular location, n = 15; subcapsular location, n = 3), whereas none of patients in RFA group had tumor at these difficult locations. The serum AFP level was also comparable between groups (Table 1).

**Table 1:** Patient demographic and clinicopathologic characteristics in SBRT group and RFA group.

Characteristics	SBRT group (n = 22)	RFA group (n = 38)	*P*
Age (years)	66.5 (35–87)	61.5 (43–77)	.243
Gender (male: female)	15: 7	31: 7	.237
Hepatitis B infection	14 (63.6)	26 (68.4)	.122
Hepatitis C infection	0	0	1.000
Presence of cirrhosis	12 (54.5)	19 (50)	.734
Comorbidity	15 (68.2)	31 (81.6)	.237
Child-Pugh Grading			
Grade A: Grade B	21: 1	35: 3	.616
Bilirubin (μmol/L)	10.6 (4.1–63.3)	16.8 (4.6–54.8)	.263
Albumin (g/L)	38 (28.2–45.8)	38.8 (20.5–50.1)	.788
Serum AFP (mmol/L)	13.1 (2.3–27246)	5.9 (1.4–12508)	.135
Size of largest tumor (cm)	4.35 (0.8–5)	1.8 (1–4)	<.001
No. of tumors treated			
Single: multiple	12: 10	18: 20	.592
Tumor at liver dome	5 (22.7)	0	.002
Perivascular tumor	15 (68.1)	0	<.001
Subcapsular tumor	3 (13.6)	0	.019
30-day mortality	0	0	1
Hospital stay (days)	38 (4–14)	1.5 (1–9)	.442
Complete ablation rate	18 (81.8)	34 (89.4)	.795

Continuous variable is expressed as median (range). Categorical variable is expressed as number (percentage). AFP = Alpha fetoprotein level, RFA = radiofrequency ablation, SBRT = stereotactic body radiotherapy.

### 3.2 Short-term outcome

There were nonspecific complaints from patients in both groups, including decreased appetite, nausea, and fatigue during first week after treatment. SBRT group had 5 patients (22.7%) developing fever after treatment, whereas RFA group had 8 patients (21%) with same symptom. These patients were treated conservatively. There was no major hepatic, renal, vascular or biliary complication among both groups of patients. There was no severe complication (Clavien-Dindo grade IIIA or above) in both groups. There was no hospital mortality after treatment in both treatment groups. CR rate at 3 months was 81.8% (18 of 22 patients) in SBRT group and 89.4% (34 of 38 patients) in RFA group. There was no statistically significant difference between two groups (*P* = .400) (Table 2).

**Table 2:** Short-term outcome of SBRT group and RFA group.

Characteristics	SBRT group (n = 22)	RFA group (n = 38)	*P*
Overall complications	5 (22.7)	8 (21)	.879
Fever	5 (22.7)	8 (21)	.879
Liver failure	0	0	1.000
Biliary complication	0	0	1.000
Intrahepatic vascular complication	0	0	1.000
Renal failure	0	0	1.000
Severe complications^∗^	0	0	1.000
Hospital mortality	0	0	1.000
Complete ablation rate	18 (81.8)	34 (89.4)	.400

Categorical variables are expressed as number of patients (percentage). RFA = radiofrequency ablation, SBRT = stereotactic body radiotherapy.

∗ Severe postoperative complication according to Clavien-Dindo grade III or above.

### 3.3 Long-term outcome

**Figure 1:**
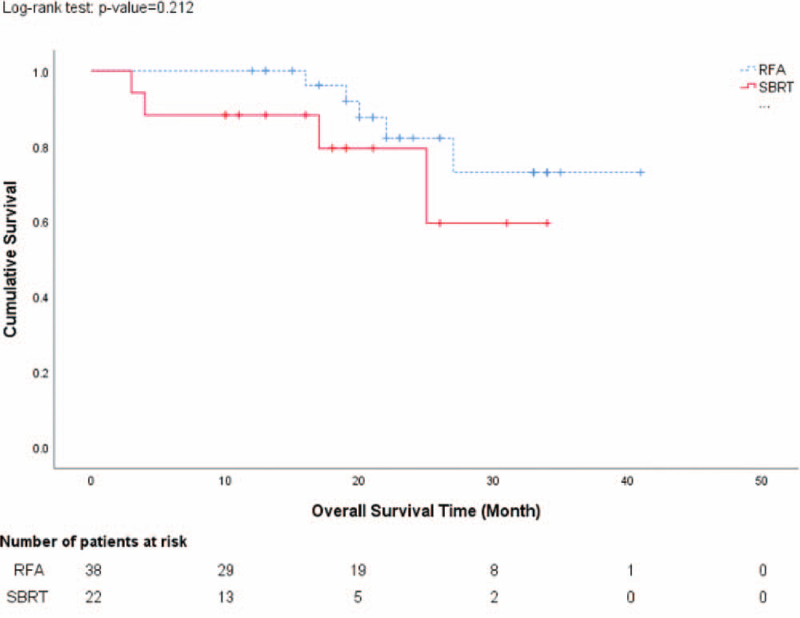
Overall survival results after SBRT and RFA for HCC. HCC = hepatocellular carcinoma, RFA = radiofrequency ablation, SBRT = stereotactic body radiotherapy.

**Figure 2:**
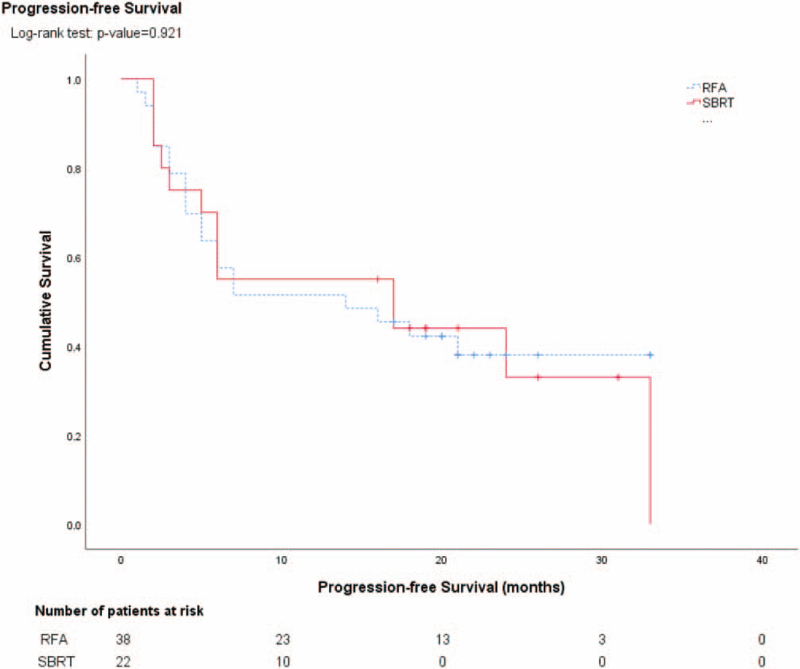
Tumor progression-free survival results after SBRT and RFA for HCC. HCC = hepatocellular carcinoma, RFA = radiofrequency ablation, SBRT = stereotactic body radiotherapy.

With median follow-up period of 26 months (range: 8–36 months), 2 patients in each group developed local tumor recurrence. The local tumor control rate was comparable between SBRT and RFA groups (90.9% vs. 94.7%). Ten of 22 patients (45.4%) in SBRT group and 20 of 38 patients (52.6%) in RFA group developed intrahepatic recurrence. There was no statistical significant difference between two groups (*P* = .592). Six patients (27.2%) in SBRT group but no patient in RFA group developed extrahepatic tumor metastasis. This might signify worse tumor biology in SBRT group than RFA group. The time to tumor recurrence was similar between groups. Recurrent tumor were more likely treated by different therapies (local ablation, TACE, systemic therapy, and radiotherapy) in SBRT group than RFA group (Table 3). The 1-year and 2-year overall survival rate was 88.2% and 85.7% in SBRT group, and 100% and 75% in RFA group, respectively. There was no statistical significant difference between two groups (*P* = .576) (Fig. 1). Causes of death included liver failure in both groups, and hepatorenal syndrome and gastrointestinal bleeding in RFA group. The 1-year and 2-year tumor progression-free survival rate was comparable between SBRT group (50% and 13.6%) and RFA group (44.7% and 7.9%) (*P* = .805) (Fig. 2).

**Table 3:** Long-term outcome of tumor recurrence in SBRT group and RFA group.

Characteristics	SBRT group (n = 22)	RFA group (n = 38)	*P*
Local tumor control rate	20 (90.9)	36 (94.7)	.566
Intrahepatic recurrence	10 (45.4)	20 (52.6)	.592
Extrahepatic recurrence	6 (27.2)	0	<.001
Intrahepatic and extrahepatic recurrence	1 (4.5)	1 (2.6)	.665
Time to tumor recurrence (months)	16 (2–33)	14 (1–33)	.934
Treatment of recurrence			
Local ablation (RFA)	3 (13.6)	3 (7.9)	
TACE	8 (36.3)	1 (2.6)	
Systemic treatment	9 (40.9)	0	
Radiotherapy	3 (13.6)	0	
Supportive care	4 (18.1)	0	

Continuous variable is expressed as median (range). Categorical variable is expressed as number (percentage). RFA = radiofrequency ablation, SBRT = stereotactic body radiotherapy, TACE = transarterial chemoembolization.

## 4 Discussion

The present study provides clinical evidence that SBRT can achieve similar oncological clearance rate as RFA for unresectable HCC and the short-term and long-term outcomes are similar between groups.

Currently, there are various modalities of local ablation therapy for HCC, including RFA, microwave ablation, cryotherapy with argon helium knife, and ethanol injection. Although RFA is recommended as a curative treatment modality for HCC in various international guidelines,^[4,16–18]^ it is not without limitations. First, it involves direct puncture of liver tumor by RF needle, which might be difficult through percutaneous route in case of unfavorable tumor location at liver dome or in close proximity to surrounding organs. Second, “heat-sink” effect might reduce RFA efficacy when the ablation is carried out near major intrahepatic vasculature. Third, there is maximal size limitation (generally 5 cm) of tumor ablation by RFA.

The application of radiotherapy for liver tumor is limited in the past because the whole liver can only tolerate low doses of irradiation (∼30 Gy), which has limited therapeutic effect. With the advancement of tumor targeting techniques in radiotherapy, focal liver irradiation (SBRT) is possible and the dose can be up to >70 Gy, which is well tolerated by patients without major complication. It is less restricted by tumor location and liver function.^[7]^ The mechanism of cytoreduction of SBRT is primarily related to direct tumor cell damage by ionizing radiation, which leads to breakdown of DNA strands, loss of clonogenicity, and subsequent cell death. SBRT makes use of hypofractionated high dose irradiation to minimize the possible repair of damaged DNA by tumor cells. The response of stroma surrounding tumors to irradiation, including tumor-associated small vessels and leukocyte populations, can also cause indirect effects on cancer cell death. In an early phase I clinical series, local tumor control rate was up to 100% with a 2-year overall survival of 60%.^[19]^ Sanuki et al^[20]^ published another series on SBRT in 185 patients with HCC. The 3-year local tumor control rate was up to 91.6% in patients receiving 40 Gy irradiation. In the present study, the maximal irradiation dose was 50 Gy given in 2 weeks’ period and the local tumor control rate was 94.9%, which concurred with results from others.

One important finding of the present study is the comparable therapeutic effects (CR rate at 3 months [81.8% vs. 89.4%], local tumor control [90.9% vs. 94.7%], 2-year overall survival [85.7% vs. 75%] and 2-year tumor progression-free survival [13.6% vs. 7.9%]) between SBRT and RFA for unresectable HCC, although the tumor size in SBRT group is larger than that of RFA group. The comparison of therapeutic efficacy between SBRT and RFA has been studied in other studies. Kim et al^[10]^ performed a recent propensity matching analysis comparing SBRT (n = 313) with RFA (n = 313). With a median follow-up of 27.7 months, 3-year local tumor recurrence rate was significantly lower in SBRT group (21.2%) than RFA group (27.9%). SBRT was superior to RFA in terms of tumor control rate in large tumor in subphrenic region and those tumor progressed after transarterial chemotherapy. Another more recent comparative study by Jeong et al^[11]^ revealed that the 4-year local tumor control rate (96.3% vs. 90.6%) and overall survival rate (70.2% vs. 71.8%) were similar between SBRT and RFA after matching. The severe complication rate was also similar between two groups (1.1% vs. 0.6%). Subsequently, Lee et al^[21]^ published a systemic review and meta-analysis on 11 studies involving 2238 patients. There was no significant difference in pooled 2-year local tumor control between SBRT group (84.5%) and RFA group (79.5%). More recently, another meta-analysis by Eriguchi et al^[13]^ has shown that SBRT was associated with similar overall survival but better local tumor control (HR 0.39) comparing to RFA when Barcelona Liver Cancer Staging factors were matched between groups. Up till now, there is only one randomized controlled trial comparing proton beam radiotherapy with RFA for recurrent HCC by Kim et al.^[22]^ The results showed that proton beam radiotherapy resulted in better 2-year local progression-free survival than RFA (92.8% vs. 83.2%). Judging from all these study results, the tumor control rate of SBRT is at least comparable, if not better, to RFA.

Irradiation-induced liver toxicity is of great concern in conventional radiotherapy to liver. It can manifest as deranged liver function, hepatomegaly, and acute cholangitis. Occasionally, there may be bystander effect of irradiation causing hepatitis B viral reactivation and liver derangement.^[23]^ One major merit of SBRT is its hypofractionated nature which can minimize long irradiation course that might lead to cytokine-induced liver toxicity. In the present study, there is no incidence of irradiation-induced liver toxicity nor major complication event in SBRT group.

Another aspect of SBRT relates to its nature of tumor microenvironment modulation and immune-modulation depending on the dosage of irradiation. These include T-cell activation and tumor-antigen presentation changes.^[24]^ These immune responses form the basis of combination therapy in which SBRT is combined with newly developed immunotherapeutic drugs in management of HCC. Besides, abscopal effect associated with SBRT has been studied recently. It involves shrinkage of tumors outside the scope of the localized treatment of tumor by SBRT. The possible underlying mechanism relates to the possible activation of immune system by ionizing irradiation against tumor cells.^[25]^

The present study is limited by the small patient number in both SBRT and RFA groups and its retrospective in nature. There is heterogeneity of tumor characteristics between two groups. Nonetheless, it provides some clinical insights regarding clinical efficacy of SBRT for HCC and it may form the basis of designing prospective study comparing SBRT with other locoregional therapy for HCC.

To conclude, this retrospective study has shown the comparable short-term and long-term outcomes between SBRT and RFT for unresectable HCC. Future prospective clinical study is needed to justify its role in patients with HCC.

## Author contributions

**Conceptualization:** Ren Ji, Kelvin K. Ng.

**Data curation:** Ren Ji, G. Wu.

**Formal analysis:** Ren Ji.

**Investigation:** Ren Ji, Wenqi Chen, Weihong Yang, Hongtao Zhu, Chi-Leung Chiang, Tiffany C.L. Wong, Feng-Ming Kong.

**Methodology:** Ren Ji, Kelvin K. Ng, Wenqi Chen.

**Project administration:** Tan-To Cheung, Chung-Mau Lo.

**Supervision:** Chung-Mau Lo.

**Writing – original draft:** Ren Ji.

**Writing – review & editing:** Kelvin K. Ng, G. Wu.
